# Improving Pneumococcal Vaccination Rates in an Inpatient Pediatric Diabetic Population

**DOI:** 10.31486/toj.22.0036

**Published:** 2022

**Authors:** Aymen Mirza, Apoorva Jagadish, Kelsey Trimble, Adijat Olanrewaju

**Affiliations:** ^1^ Department of Pediatrics, Ochsner Louisiana State University Health–Shreveport, Shreveport, LA; ^2^ Department of Pharmacy, Ochsner Louisiana State University Health–Shreveport, Shreveport, LA; ^3^ Department of Hospital Medicine, Ochsner Louisiana State University Health–Shreveport, Shreveport, LA

**Keywords:** *Diabetes mellitus*, *pneumococcal vaccines*, *vaccination*

## Abstract

**Background:** Diabetes is an immunocompromising condition, and diabetic children should receive the 23-valent pneumococcal polysaccharide (PPSV23) vaccine as part of their preventive care because of their increased risk for invasive pneumococcal disease. This recommendation is often not followed, however, and at our institution, we discovered that a factor limiting vaccine administration was lack of knowledge about the recommendation among residents.

**Methods:** Our objective with this quality improvement initiative was to improve pneumococcal vaccination rates among the inpatient pediatric diabetic population to 70% in 6 months. Three education and awareness initiatives were conducted during the postintervention period of March 2021 to August 2021 at St. Mary Medical Center in Shreveport, Louisiana. All pediatric diabetic patients from age 2 to 18 years who were admitted to the inpatient general pediatrics or critical care services were included. The primary outcome was vaccination with PPSV23.

**Results:** We studied 63 pediatric patients with a mean age of 12.7 years. The vaccination ordering rate during the 6 months prior to the implementation of the quality improvement initiatives was 41%. In the 6 months postintervention, the overall vaccination ordering rate improved to 81%. During data collection, however, we discovered that even though the residents were assessing for vaccine eligibility and ordering the vaccines, not all vaccines were administered prior to discharge. In the preintervention period, the overall vaccine administration rate was 27%, improving to 42% in the postintervention period.

**Conclusion:** Simple interventions that included resident education, development of a smart phrase in the electronic medical record, and liaison with pharmacy led to an increase in the pneumococcal vaccination ordering rate for pediatric patients with diabetes. However, we did not anticipate that the vaccination ordering and administration rates would be different when we initiated the project and had therefore focused our interventions on resident education only. Our discovery of the difference between vaccination ordering and vaccination administration helped identify 2 other areas for improvement: nursing education and additional improvement of the electronic medical record.

## INTRODUCTION

*Streptococcus pneumoniae* remains a leading cause of serious illness, including bacteremia, meningitis, sinusitis, otitis media, and pneumonia among children and adults worldwide.^1^ Pneumococcal pneumonia results in 900,000 cases and 400,000 hospitalizations annually in the United States.^2^ The introduction of the pneumococcal vaccination series in 2010 with 4 doses administered at 2, 4, 6, and 12 to 15 months of age led to a significant reduction of invasive pneumococcal disease in healthy populations. However certain high-risk groups need surveillance and interventions.^3^

Diabetes is a recognized immunocompromising condition, and diabetic children should receive the 23-valent pneumococcal polysaccharide (PPSV23) vaccine as part of their preventive care because of their increased risk for invasive pneumococcal disease. The Advisory Committee on Immunization Practices (ACIP) and the Centers for Disease Control and Prevention (CDC) recommend that 1 dose of PPSV23 be administered to diabetic children aged 2 years and older at least 8 weeks after the child has received the final dose of 13-valent pneumococcal polysaccharide-protein conjugate vaccine (PCV13).^4^

This recommendation is often not followed even though the vaccination is considered essential preventive care for diabetics. At our institution, we discovered that a factor limiting vaccine administration was lack of knowledge about the recommendation among residents. We initiated a quality improvement project to increase the pneumococcal vaccination rate among pediatric diabetic patients admitted to the inpatient service to 70% in 6 months.

## METHODS

St. Mary Medical Center in Shreveport, Louisiana, is an academic tertiary center with 23 general pediatric beds and 8 pediatric intensive care unit (PICU) beds. A total of 24 categorial pediatrics residents and 20 internal medicine-pediatrics residents rotate through the general pediatrics and PICU services. Residents play an integral role in patient care. Epic (Epic Systems Corporation) is the electronic medical record (EMR) system.

### Interventions

We conducted a literature review to determine the current guidelines for administration of PPSV23 to diabetic patients.^4^ A multidisciplinary team consisting of 1 pediatric hospitalist faculty member, 2 categorical pediatrics residents, and 1 pediatric pharmacist was formed to review compliance with these guidelines at our institution and develop an intervention to improve compliance.

The initial intervention was implemented in March 2021. CDC guidelines for pneumococcal vaccination of pediatric patients with diabetes were shared with residents once at morning conference via an oral presentation followed by discussion. An EMR smart phrase was created to ensure uniform documentation that a pneumococcal vaccination review was done for all diabetic patients admitted to the service and that the vaccine was offered to patients who had not been vaccinated. Once diabetic patients were identified, their immunization records were reviewed to determine their eligibility for PPSV23. If a patient was determined eligible, consent for vaccination was sought from the parents and the EMR was documented to note if the vaccine was ordered or if it was refused. A pediatric clinical pharmacist participates in multidisciplinary rounds every day, and one of the pharmacist's roles is to review immunization records for all patients. Therefore, the pharmacist also helped remind and prompt physicians to order vaccines for eligible patients.

A second intervention was implemented in April 2021 and included a mass email to all residents about the guidelines and eligibility criteria for pneumococcal vaccination. This intervention reached all 44 residents as the email was sent to their individual email accounts. Fliers were also posted in resident workstations to serve as visual reminders.

The third intervention occurred in July 2021 and involved sending monthly reminder emails to residents on inpatient services for the remaining 2 months of the study. These emails included the vaccination guidelines and criteria and reached the 10 residents rotating through inpatient services for those months.

Baseline data were collected by reviewing medical records from the 6 months prior to the initiation of the project (September 2020 to February 2021). Inpatient diabetic admissions to the pediatrics ward and PICU after the intervention (March 2021 to August 2021) were identified, and the patients’ pneumococcal vaccination records were reviewed using the Louisiana Immunization Network System (LINKS) web portal.^5^ This quality improvement initiative was approved by the Louisiana State University Health–Shreveport Institutional Review Board.

### Analysis

The outcome measure was the PPSV23 vaccination rate among inpatients with diabetes aged 2 to 18 years following resident education. Random medical records were audited throughout the study period to ensure that the EMR smart phrase was being used and that documentation was adequate.

Medical records were reviewed monthly to assess the number of diabetic patients admitted and whether pneumococcal vaccination was ordered and administered prior to discharge. The vaccination ordering rate for each month was calculated by using the total number of eligible diabetic patients as a denominator and the number of vaccines offered as the numerator. Data for vaccines offered were obtained from the order history in the EMR. The vaccination administration rate for each month was calculated by using the total number of eligible diabetic patients as a denominator and the number of vaccines administered as the numerator. Statistical analysis was performed with Excel (Microsoft Corporation).

## RESULTS

A total of 63 medical records were reviewed from September 2020 to August 2021. The mean age of the patient population was 12.7 years. Eight of the 63 patients were already vaccinated and were excluded from analysis.

Prior to implementing the interventions, 9 of 22 eligible patients admitted from September 2020 to February 2021 had the vaccine ordered for them, for a vaccination ordering rate of 41% (Table 1).

**Table 1.** Preintervention (September 2020 to February 2021) Data by Month and Overall

Month	Diabetic Patients Admitted, n	Vaccinated Patients, n	Unvaccinated Patients, n	Vaccines Offered, n	Vaccination Ordering Rate, %	Vaccines Administered, n	Vaccination Administration Rate, %
September	7	2	5	2	40	1	20
October	5	1	4	1	25	1	25
November	1	0	1	1	100	0	0
December	2	0	2	0	0	0	0
January	8	1	7	3	43	2	29
February	3	0	3	2	67	2	67
Overall	26	4	22	9	41	6	27

**Figure 1.**
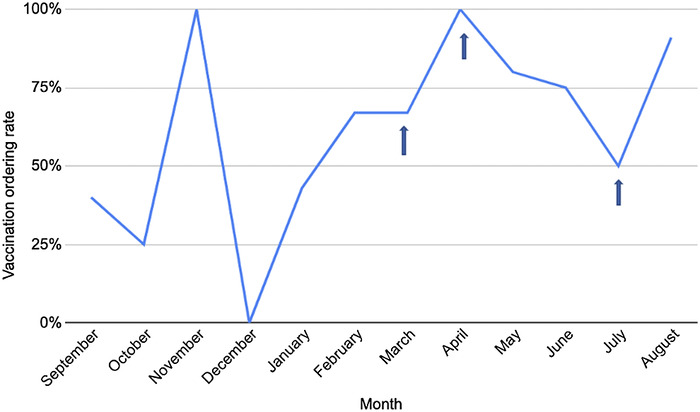
Vaccination ordering rate by month during the entire study period (September 2020 to August 2021). Arrows indicate the months during which the 3 interventions were implemented.

After the first intervention in March 2021, the vaccination ordering rate remained consistent with February at 67% (Figure 1). In April 2021, the second intervention took place and resulted in a 100% vaccination ordering rate (Table 2). In the following months, the vaccination ordering rate dropped, so the third intervention was implemented in July 2021 and led to a 91% vaccination ordering rate in August 2021.

**Table 2.** Postintervention (March 2021 to August 2021) Data by Month and Overall

Month	Diabetic Patients Admitted, n	Vaccinated Patients, n	Unvaccinated Patients, n	Vaccines Offered, n	Vaccination Ordering Rate, %	Vaccines Administered, n	Vaccination Administration Rate, %
March	6	0	6	4	67	4	67
April	5	0	5	5	100	3	60
May	5	0	5	4	80	0	0
June	7	3	4	3	75	1	25
July	2	0	2	1	50	0	0
August	12	1	11	10	91	6	55
Overall	37	4	33	27	81	14	42

During the postintervention time period, the overall vaccination ordering rate improved to 81% (27 of 33 eligible patients) from March 2021 to August 2021 compared to the 41% vaccination ordering rate prior to intervention.

**Figure 2.**
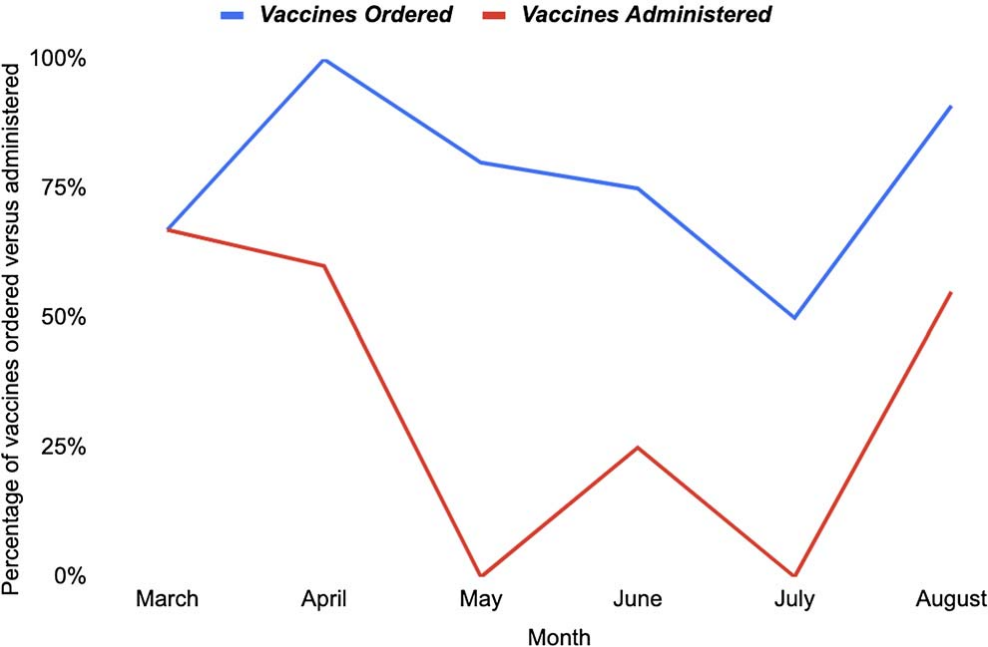
**Percentage of vaccines ordered vs vaccines administered per month during the postintervention period of March 2021 to August 2021**.

However, we noticed a discrepancy between the number of vaccines ordered by residents and the number administered by nursing staff per month, so the vaccination ordering rates we had calculated were not a true reflection of vaccination administration rates. We repeated the data analysis using number of vaccines administered per month as the numerator. Tables 1 and 2 and Figure 2 show the vaccination administration rates. In the preintervention period, the overall vaccination administration rate was 27%. In the postintervention period, the overall vaccination administration rate was 42%.

## DISCUSSION

ACIP issued guidelines for PPSV23 vaccination in 2010, identifying the following comorbidities as high risk: chronic pulmonary diseases, sickle cell disease, cochlear implants, congenital or acquired asplenia, chronic kidney diseases, immunosuppressive states, and diabetes.^6^ Diabetes is included in this list as patients with diabetes are 1.4 times more likely to develop community-acquired pneumonia and up to 4.6 times more likely to acquire invasive pneumococcal disease than the general population.^7^ Vaccination with PPSV23 can help reduce the risk. A retrospective study conducted by Kuo et al determined that PPSV23 vaccination was effective in reducing the risks of invasive pneumococcal disease and respiratory failure in diabetic adults.^8^

Despite the strong recommendations, barriers to vaccination exist in high-risk populations. Trovato et al determined in a retrospective review that the 3 principal reasons for nonvaccination of patients with a medical indication for vaccination with PPSV23 were (1) vaccination was not addressed during the visit, (2) the provider misclassified high-risk patients as low-risk patients for infection, and (3) the patient refused the vaccine.^9^ These data suggest that most barriers are linked to lack of knowledge among providers and that if providers were better advocates, rates of vaccination could be higher. Page et al demonstrated that pharmacist education increased PPSV23 vaccination rates at 3 grocery chain pharmacies, and the primary barrier to vaccination they identified was that patients wanted to discuss the recommendation with their provider.^10^

Using this evidence, we hypothesized that resident education would lead to better vaccination rates at our institution, so our primary intervention was educating residents about the current recommendations. Our initial results showed that resident education resulted in vaccines being ordered more frequently for eligible patients. Based on this data, we were able to achieve our goal of obtaining a vaccination ordering rate >70%.

However, we discovered a large difference between our vaccination ordering and administration rates. We did not anticipate this issue when we initiated the project and had therefore focused our intervention on resident education only with the expectation that if residents knew they needed to order the vaccine, the patients would get the vaccine except when the parents refused. However, our experience was contrary, and we realized that despite orders being placed, patients were not getting vaccines.

Discussions with nursing management and hospital administration helped identify the sources of this deficit. In some cases, the vaccine was ordered without a specified time of administration, so the EMR did not alert the nurses that the vaccine was due. In other cases, residents ordered more than one type of pneumococcal vaccine (PCV13 and PPSV23) at the same time, leading to confusion and lack of execution. Further, ordering the vaccine to be administered “at discharge” leads to logistic delays as the vaccine order must be verified and released by the pharmacists who then send the vaccine vial to the ward for administration. This process led to delays in discharge when both nursing and provider teams were pressed from hospital administration for timely discharges. Therefore, to avoid these delays, some vaccine orders were overridden.

During further discussion with nursing leadership, we discovered that the vaccine order sometimes appears as a PRN (as needed) order in the nurses’ workflow. Orders can be inadvertently missed when they do not show up as due, when no time of administration is specified, or when the order disappears once a discharge medication reconciliation has been completed and discharge orders placed. Most of these errors were attributable to the intrinsic mechanism of the EMR. This discovery helped identify other areas for improvement: nursing education and improvement in the EMR.

During the data analysis phase of this project, we realized that the nonvaccination problem is multidisciplinary and that the effect of our interventions decreased over time. This knowledge identified the need for a more sustainable and reliable solution. We are now advocating for a clinical decision tool in our EMR that will prompt providers for an intervention based on the standard of care. A classic example of a well-structured clinical decision tool is the one for a patient with the diagnosis of stroke: a pop-up box reminds the provider to screen for anticoagulants. At our institution, we have a clinical decision tool for patients with sickle cell, a pop-up box indicting their eligibility for pneumococcal vaccination that prompts the provider to verify their immunization status in LINKS.

Based on the results of our study, we have strong grounds to advocate for a similar clinical decision tool in our EMR for our pediatric diabetic population as well as other eligible patients. Our next step is to present our data to hospital administration and initiate conversations with the information technology department on how to build and implement this tool. Once functional, this clinical decision tool will be a sustainable intervention that does not need to be repeated each time a new team comes on service. The end goals are improved patient care and better compliance with guidelines for our diabetic population.

## CONCLUSION

Simple interventions that included resident education, development of a smart phrase in the EMR, and liaison with pharmacy led to an increase in the pneumococcal vaccination ordering rate for pediatric patients with diabetes. However, an increase in the vaccination administration rate can be attained by including nursing staff education. Our next step is to advocate for changes in the EMR system to flag qualifying patients and prompt the provider to order and administer the vaccine.

## Notes

*This article meets the Accreditation Council for Graduate Medical Education and the American Board of Medical Specialties Maintenance of Certification competencies for Patient Care and Medical Knowledge.*
